# Mineral Teeth

**Published:** 1850-07

**Authors:** Solyman Brown


### ARTICLE V.

### *Mineral Teeth.* By Solyman Brown, M. D.

The triumph of art in restoring lost members of the human physical organism, is nowhere exemplified in so signal a manner, capable of general and useful application, as in the invention and perfected manufacture of artificial mineral teeth.

Without depreciating the advantages of artificial eyes, hair and legs, to those who have lost them, we must rank the substitution of beautiful artificial masticators for those of nature which have been lost by disease or accident, as pre-eminent above them all; and this, for several reasons. Of these we have time at present to enumerate only a part; but even a cursory consideration of these few reasons will abundantly confirm our position.

Artificial eyes, and even hair, may indeed, and often do, when well adapted, confer advantages by no means to be neglected, especially by those individuals who can afford some pecuniary expenditure for the sake of personal appearance. Artificial legs not unfrequently confer upon the mutilated soldier or citizen, the coveted ability of walking without crutch or staff. But we have said that several reasons combine to confer greater utility to entire humanity upon artificial substitutes in the dental arch, than upon any or all of these.

In the first place, a very large proportion of the human family who arrive at mature age, may be benefited essentially by artificial teeth, both for use and ornament, whilst only a comparatively small portion ever stand in need of substituted eyes, hair or limbs.

In truth such are the generally diffused refinements of the present civilization, especially in the cities, towns and villages of these prosperous States, and to a great extent even in the country, that artificial teeth when required for either one or the other of the forenamed reasons, have taken their position among the very necessaries of life.

In the second place, the use of other substituted parts of the human frame, is not so vital in its character as to involve both health and longevity, as does the substitution of a masticating apparatus, when that of nature has been destroyed. In this lies essentially the paramount importance of the present improvements in artificial teeth; and it is this which leads me to offer a few remarks in this place, on their manufacture.

Its progress, though rapid, has not been of long continuance; and though Europe has the credit of beginning the work, it is the pride of American artists that they have advanced it greatly beyond its transatlantic condition.

As evidence of this, it may be allowable to state that the writer of this article has received an order by the last Atlantic steamer, through a London House, for Stockton's American Teeth, from the British dominions in New South Wales, a position on the earth almost exactly antipode to the place of their manufacture. This last circumstance is not less remarkable than that the order should not have been sent to Europe, for the reason that it could not be filled there to the satisfaction of European colonies in the opposite hemisphere. And all this was done at the additional expense of heavy duties in the British customs, paid by English subjects. What a compliment is here to this particular branch of American production!

In addition to the foregoing fact, it may be stated here, that orders come continually from almost every country in Europe, even from London and Paris themselves, where this species of manufacture was commenced, long before its introduction to these States.

Inasmuch as Dr. Harris and other popular authors, have given statements of the rise and progress of this manufacture in their recent works, I shall omit its merely historical details on this occasion, for the purpose of speaking more at large of its distinctive features and characters in the three countries which gave it birth.

Of these, the first may be distinguished as the French; the second as the English; and the third, as the American manufacture.

The French was the first in time, but last in elegance and perfection of workmanship. It lacked not the qualities of hardness and unchangeableness under the blowpipe, but it lacked translucency and elegance of form.

The most transparent and elegant of all mineral teeth, may be denominated the English, because they were first manufactured either in England itself or its Canadian dependency, and perhaps, furthermore, because the English dentists have persevered in their use, after those of other nations have found it necessary to reject them.

The reasons for this rejection are, that although in beauty of form and elegance of texture they stand unrivalled, they lack the indispensable quality of resisting high degrees of heat, without which no artificial teeth can be inserted in the mouth to permanent advantage. Hence the truly beautiful fabric of Mr. Ash, of London, can be justly regarded as nothing more nor less than a splendid failure, because it actually fails to solve the practical problem proposed in the maufacture of mineral teeth. Robert Fulton may just as properly have claimed the honor of impelling boats by steam without the use of fire, as that any man should claim that he has met the demands of the market by producing mineral teeth which will not sustain uninjured the necessary degree of heat to solder gold.

The American teeth, in contradistinction to both the others, combine beauty with utility. They gratify the taste by possessing the splendor of the pearl, whilst they resist the fire with the intrepidity of the salamander. This was "the consummation so devoutly to be wished," and to which the untiring exertions of S. W. Stockton triumphantly led the way. Although not the first individual who attempted the manufacture of mineral teeth, he was undeniably the first to demonstrate to the profession that America could raise them to a perfection never yet reached in the old world. It is now nearly a quarter of a century since Mr. Stockton established this fact upon a basis that has never yet been shaken; and with the exception of those individuals who have profited by his labors, and also Mr. James Alcock, of New York, who has done himself great credit by his honorable success as well as by his gentlemanly manners, Mr. Stockton continues to this period to command the market of the world.

It now remains that he should remunerate the profession which has raised him to fame and fortune, by completing the work which he has thus honorably begun. If the hero of Chapultepec had reposed contentedly upon the laurels of his suburban victory, he would never have marshalled his troops in the Plaza of Mexico. Let Mr. Stockton remember this, and renew his labors. It now remains for him to finish the work which he has thus honorably begun, by so perfecting his *forms*, and so arranging his *shades*, as to place it beyond the power of rivalry to excel him. In this direction he may yet perform a work for which the dental profession and those who are benefited by their labors, shall transmit his eulogy to remote posterity; even to that era of the world, should it ever come, when the wisdom and the virtue of the human race shall restore that Eden of health and happiness which man has lost.

We have reason to know that much as remains to be done on this subject, Mr. Stockton is neither idle nor unambitious in its performance. Family afflictions have indeed for a time paralyzed his ambition, but we trust the sorrows of the past will be greatly alleviated by joys to come, so that the bard of Avon may truly say, "Richard is himself again."

In making these remarks for the pages of the American Journal of Dental Science, of which I was once an editor, and from which, as a contributor I had supposed myself forever separated, I cannot wish to conceal the fact, that I have placed myself in a position which makes me an interested party in the manufacture of which I speak. If the manufacture prospers, so may I, who have become its agent.

But I regard my position between the manufacturer and the profession, as one which enables me to be useful to them both. It is my interest and theirs, that the teeth made and bought and sold, should be of such a quality as to leave nothing to be desired. It is the interest of all of us that they be sold at proper prices, and I have already so far used my influence as to induce Mr. Stockton to reduce his prices for teeth with gums, from twenty-five to twenty cents. I have done this not for the purpose of underselling others; nor yet for the purpose of reducing the aggregate of Mr. Stockton's profits; much less for the purpose of decimating my own commissions; but solely and exclusively because I regard the price now set as more just and equitable.

In relation to the other kinds of teeth, viz: all those without gums, I should advise a tariff somewhat different from the present—namely—ten cents for pivot teeth, twelve and a half cents for incisor plate teeth, and fifteen cents for molars.

With this tariff the ambition of the manufacturer should be directed to the improvement of his stock; and in this should consist the rivalry of competition. It would be better for the dentist as well as the public, that the teeth used should be worth the advanced prices at all times, than that they should be of inferior quality at smaller rates. Let the manufacturers have such a tariff of prices as will enable them greatly to improve their moulds, multiply their varieties, and diversify their colors. Much indeed has been already done in all these respects, but still more remains to be accomplished. It shall be my ambition as long as I stand thus between the two parties—the manufacturer and the dentist, to do every thing in my power to promote the interest of both. Considerable practical experience during the last quarter of a century, has taught me the interests of both, and to these interests, as well as to my own, I intend to be faithful. I wish to put in practice one of the central precepts of christianity, that he who best performs his devotions towards God, is he who exercises most faithfully the duties of his office towards his fellow men. With these observations which are addressed particularly to my old friends and fellow-laborers in the dental profession, to which it gives me great pleasure to believe, that in former years, I have done some service, I invite them to coöperate with my efforts in attempting to do something more in years yet to come.

